# *In Vitro* BBB triculture assay and preliminary computational model development to predict brain exposure

**DOI:** 10.3389/ftox.2026.1781588

**Published:** 2026-03-10

**Authors:** Zhuangyan Monica Xu, James P. Sluka, Charlie C. Zhang, Gregory Knipp

**Affiliations:** 1 Department of Industrial and Molecular Pharmaceutics, College of Pharmacy, Purdue University, West Lafayette, IN, United States; 2 Biocomplexity Institute, Intelligent Systems Engineering, Indiana University, Bloomington, IN, United States

**Keywords:** apparent permeability, blood-brain barrier, CNS exposure prediction, efflux ratio, high throughput toxicokinetics in R, HTTK-R, in vitro-in vivo, IVIVE

## Abstract

**Introduction:**

Neurotoxicity is a critical liability for many environmental pollutants. Current in vitro neurotoxicity screens rely on direct exposure of cultured neurons to xenobiotics, often at exceeding physiologically relevant levels due to the restrictive nature of the blood -brain barrier (BBB). This limitation reduces the accuracy of central nervous system (CNS) exposure predictions.

**Methods:**

To address this limitation, we have developed a novel human in vitro direct-contact triculture BBB model that more closely mimics the in vivo barrier. The triculture is formed by layering primary astrocytes, primary pericytes, and then brain microvessel endothelial cells (BMECs, HBEC-5i) in direct contact, increasing the restrictive nature of tight junctions and allowing cell -cell signaling that mimics the configuration found in the in vivo BBB. Using this model, we quantified the apparent bidirectional permeability (P_app_) of more than 50 compounds, including environmental pollutants and CNS drugs, primarily by paracellular, passive transcellular and transporter-mediated pathways, to help develop a risk of exposure. In parallel with our in vitro BBB model, we are using the high-throughput toxicokinetics (HTTK) R library developed by the U.S. Environmental Protection Agency (EPA) as our model to predict brain exposure.

**Results:**

The triculture model demonstrated enhanced tight junction organization and increased efflux transporter expression compared with endothelial monocultures, indicating an improved barrier phenotype. Using our measured bidirectional Papp values, calculated efflux ratios, and EPA physiologically based pharmacokinetic (PBTK) reference data for compound parameters, we are developing predictions of toxicant accumulation in the brain parenchyma after chronic exposure in steady state.

**Discussion:**

Integration of *in vitro* BBB permeability measurements with toxicokinetic modeling provides a physiologically relevant approach to predict CNS exposure and neurotoxicity risk. Through the development of this model, we postulate that future investigators could simply perform in vitro BBB permeability studies to determine the relative risk of potential brain accumulation and the risk of neurotoxicity.

## 1 Introduction

Neurotoxicity is a major liability associated with both environmental pollutants and therapeutic agents, raising significant concerns for public health and regulatory agencies (Walsh et al., 2024). Exposure to industrial and environmental chemicals has been linked to adverse neurodevelopmental and neurodegenerative outcomes, which has prompted regulators to prioritize neurotoxicity risk assessment in chemical safety evaluations (Bharal et al., 2024). Likewise, many drug candidates targeting the central nervous system (CNS) fail in late stage development, often due to an inability to effectively penetrate the blood-brain barrier (BBB) (Pardridge, 2005; Bhowmik et al., 2015; Neumaier et al., 2021). These challenges underscore the need for more human-relevant models to evaluate neurotoxic potential and CNS drug delivery early in the risk assessment and development process.

However, current *in vitro* neurotoxicity screening approaches typically do not incorporate a functional BBB (Juberg et al., 2023). As a result, test systems often employ xenobiotic concentrations that greatly exceed realistic brain levels, as there is no barrier to restrict or modulate chemical entry into neural cells. The BBB, composed of brain microvessel endothelial cells (BMECs) interconnected by tight junctions and supported by astrocytes and pericytes, functions to restrict the transport of substances from the bloodstream to the brain parenchyma (Daneman and Prat, 2015; Juberg et al., 2023). Without an analogous barrier *in vitro*, toxicity-based assays using neurons alone can overestimate the neurotoxic potency of a compound or mischaracterize its safety margins, because they cannot account for pharmacokinetic constraints of the BBB on CNS exposure. In fact, the lack of representation of BBB in traditional neurotoxicity tests is widely recognized as a gap that hinders the accurate *in vitro* to *in vivo* extrapolation (IVIVE) for neurotoxic risk (Illa et al., 2025).

To address this critical gap, we previously developed a physiologically relevant *in vitro* direct-contact triculture BBB model (Lubin and Knipp, 2021) and integrated it into a toxicokinetic framework. The *in vitro* BBB model comprises human primary astrocytes, primary pericytes and BMECs (HBEC-5i) cultured in a layered direct contact configuration that structurally and functionally recapitulates the architecture of the native neurovascular unit (NVU) (Lubin and Knipp, 2021). This direct contact triculture arrangement facilitates cell-cell signaling and synergistic interactions responsible for the restrictive barrier phenotype observed *in vivo*. Moreover, as federal agencies put forth initiatives to reduce the use of animals in research (*e.g.,* FDA Modernization Act 3.0), an increasing emphasis is now placed on establishing physiological relevancy in cell based screening models.

In this study, we applied our *in vitro* BBB triculture model to a panel of structurally and functionally diverse compounds of environmental, regulatory and pharmacological relevance. The test set encompassed a diverse panel of CNS drugs, environmental toxicants, industrial chemicals, and emerging compounds of regulatory concern. By challenging the model with this chemically diverse set, we aimed to rigorously evaluate its ability to discriminate BBB permeability profiles across compounds with different physicochemical properties and transport characteristics. Bidirectional permeability measurements provide a quantitative framework for distinguishing passive diffusion from transporter-mediated efflux at the BBB and have been shown to yield efflux ratios that relate *in vitro* BBB transport behavior to CNS drug disposition (Summerfield et al., 2007). In this study, bidirectional apparent permeability $\left(P_{\mathrm{app}}\right)$ values and efflux ratios were quantified for each compound using our *in vitro* direct contact BBB model, generating a dataset of compound-specific BBB transport metrics. We then integrated the experimental data with a toxicokinetics simulation platform (HTTK) to improve IVIVE predictions of chronic brain exposure. The HTTK framework, implemented in R, uses human *in vitro* ADME data and generic physiologically based pharmacokinetic models to estimate chemical distribution and concentrations in the body (Pearce et al., 2017). The HTTK package includes a multi-compartment physiologically based toxicokinetic model (PBTK), but unfortunately that model does not include a brain compartment. Here, we extend the HTTK model by using the measured BBB permeability apparent permeability coefficients, $P_{\mathrm{app}}$ , for each chemical as a scaling parameter, to estimate the brain penetration aspect of the toxicokinetic simulation. By doing so, we accounted for compound-specific BBB transfer rates when predicting both acute and chronic exposure in the CNS. This integrated approach enables a more realistic translation of *in vitro* neurotoxicity assay concentrations to *in vivo* brain-equivalent doses, improving confidence in hazard prediction and risk assessment.

## 2 Materials and methods

### 2.1 Materials

Primary human brain astrocytes, vascular pericytes, and their corresponding growth media (astrocyte, pericyte, and endothelial) were acquired from ScienCell™ Research Laboratories (Carlsbad, CA, United States). HBEC-5i cells, an immortalized BMEC human brain cell line, was obtained from ATCC (Manassas, VA, United States). Transwell® inserts (12 mm diameter, 0.4 μ m pore size), type I rat tail collagen, Matrigel®, and T75 flasks were sourced from Corning (Corning, NY, United States). Hank’s Balanced Salt Solution (HBSS) was purchased from Gibco (Carlsbad, CA, United States). All test compounds listed in Table 1, along with paraformaldehyde, dimethyl sulfoxide (DMSO), and poly-L-lysine (PLL), were supplied by MilliporeSigma (St. Louis, MO, United States).

**TABLE 1:** BBB Triculture compounds and assay results.^a^

​	​	Apparent Permeability ( $P_{app}$ )		​	​
​	​	(x10^−6^ cm/s) ( ± s.d.)		Efflux	Permeability
CASRN	Compound^b^	$P_{\mathrm{app}}^{AB}$	$P_{\mathrm{app}}^{BA}$	Ratio^c^	Rate
22083-74-5	DL-Nicotine	0.08 ± 0.01	0.08 ± 0.04	0.98	Slow
89-25-8	Edaravone	0.41 ± 0.13	0.25 ± 0.16	0.61	Slow
52-53-9	Verapamil	0.57 ± 0.02	1.0 ± 0.14	1.75	Slow
88-99-3	Phthalic acid	0.75 ± 0.19	0.59 ± 0.05	0.79	Slow
1222998-36-8	Torin 1	2.2 ± 0.07	1.3 ± 0.6	0.56	Moderate
103-90-2	Acetaminophen	3.7 ± 0.64	2.3 ± 0.5	0.62	Moderate
19216-56-9	Prazosin	3.7 ± 0.8	7.0 ± 1.8	1.88	Moderate
53230-10-7	Mefloquine	4.7 ± 0.1	36.9 ± 4.4	7.81	Moderate
50-23-7	Hydrocortisone	4.8 ± 2.5	1.3 ± 0.6	0.26	Moderate
259793-96-9	Favipiravir	5.2 ± 2.4	4.4 ± 1.7	0.83	Moderate
535-80-8	3-Chlorobenzoic acid	5.6 ± 0.6	3.1 ± 0.6	0.55	Moderate
123-31-9	Hydroquinone	7.2 ± 3.5	10.0 ± 3.9	1.38	Moderate
84-66-2	Diethyl phthalate	7.7 ± 1.1	7.2 ± 1.6	0.94	Moderate
88321-09-9	Aloxistatin	8.1 ± 0.8	6.6 ± 0.4	0.81	Moderate
2628280-40-8	Nirmatrelvir	8.5 ± 0.1	6.9 ± 0.2	0.81	Moderate
551-16-6	6-Aminopenicillic acid	10.6 ± 1.6	16.3 ± 1.9	1.54	Moderate
298-46-4	Carbamazepine	12.2 ± 1.3	10.9 ± 0.4	0.90	Fast
60-92-4	Adenosine 3′,5′-cyclic monophosphate (cAMP)	12.4 ± 2.8	13.0 ± 2.8	1.04	Fast
15686-71-2	Cephalexin	12.7 ± 3.3	25.8 ± 2.5	2.03	Fast
5786-21-0	Clozapine	13.4 ± 1.2	12.0 ± 1.5	0.89	Fast
579-75-9	2-Methoxybenzoic acid	13.4 ± 1.5	10.5 ± 0.6	0.78	Fast
2492423-29-5	Molnupiravir	13.4 ± 0.1	10.7 ± 0.9	0.80	Fast
51-28-5	2,4-Dinitrophenol	13.5 ± 0.7	11.3 ± 1.5	0.84	Fast
2798-05-2	4,4′-Methylenebis(phenyl isothiocyanate)	13.5 ± 3.9	9.8 ± 3.1	0.73	Fast
120-83-2	2,4-Dichlorophenol	13.6 ± 2.2	13.5 ± 3.0	0.99	Fast
54910-89-3	Fluoxetine	14.4 ± 4.7	20.1 ± 3.6	1.4	Fast
57-41-0	Phenytoin	15.1 ± 2.8	14.6 ± 2.2	0.96	Fast
118-92-3	Anthranilic acid	15.9 ± 1.6	12.2 ± 0.77	0.77	Fast
58-08-2	Caffeine	16.6 ± 1.7	10.6 ± 2.3	0.64	Fast
na	BisMetPtNH2^d^	17.4 ± 1.5	22.9 ± 3.5	1.31	Fast
67-20-9	Nitrofurantoin	18.3 ± 0.6	23.5 ± 1.3	1.28	Fast
414864-00-9	Belinostat	18.8 ± 3.6	0.07 ± 0.01	0.004	Fast
4044-65-9	1,4-Phenylene diisothiocyanate	20.0 ± 3.0	6.6 ± 1.8	0.33	Fast
105650-23-5	2-Amino-1-methyl-6-phenylimidazo[4,5-b]pyridine	20.7 ± 6.0	14.6 ± 3.0	0.70	Fast
54-64-8	Thimerosal	20.8 ± 2.2	19.4 ± 1.6	0.93	Fast
4685-14-7	Paraquat	21.9 ± 3.7	20.6 ± 7.0	0.94	Fast
80-05-7	Bisphenol A	22.9 ± 6.7	28.5 ± 1.8	1.24	Fast
101-26-8	Pyridostigmine Bromide	23.0 ± 3.4	14.0 ± 7.6	0.61	Fast
987-78-0	Cytidine 5′-diphosphocholine	25.9 ± 3.0	37.5 ± 0.7	1.45	Fast
118-91-2	2-Chlorobenzoic acid	28.7 ± 5.5	26.6 ± 3.8	0.93	Fast
2474-72-8	Hydroxyquinone	29.9 ± 3.4	9.3 ± 0.5	0.31	Fast
636-00-0	6-Hydroxydopamine hydrobromide	31.3 ± 3.9	10.8 ± 1.9	0.34	Fast
na	BisMetPt^*d*^	33.9 ± 4.0	36.0 ± 2.6	1.06	Fast
6998-60-3	Rifamycin SV	45.9 ± 2.1	29.9 ± 7.6	0.65	Fast
150-76-5	Methoxyphenol	54.8 ± 11.1	37.6 ± 10.2	0.69	Fast
10265-92-6	Methanidophos	56.1 ± 7.7	18.3 ± 11.9	0.33	Fast
99-66-1	Valproic acid	> 500	117. ± 14	< 0.24	Very fast
43121-43-3	Triadimefon	> 500	149. ± 14	< 0.30	Very fast

^a^ Compounds that were insufficiently soluble (4 total), or otherwise failed (8 total) in the BBB, assay are listed in tables S2 and S3 in the Supplementary Material.

^b^ Compounds are sorted by ascending $P_{\mathrm{app}}^{AB}$

^c^ $P_{\mathrm{app}}^{BA}$ / $P_{\mathrm{app}}^{AB}$ .

^d^ See (Behymer et al., 2024) and the Supplementary Material for the structures of these compounds.

For immunofluorescence staining, primary antibodies specific to occludin (FITC-conjugated), claudin-5 (Alexa Fluor™ 488), zonula occludens-1 (ZO-1), multidrug resistance-associated protein 2 (MRP2), and P-glycoprotein (P-gp), as well as the Alexa Fluor™ 488-conjugated goat anti-mouse IgG secondary antibody, were purchased from Invitrogen (Waltham, MA, United States). Nuclear and F-actin cytoskeletal staining reagents included DAPI and ActinRed™ 555 ReadyProbes™ (rhodamine phalloidin), also from Invitrogen. Additional materials used for microscopy, including Permount™ mounting medium, glass slides, and coverslips, were also purchased from Invitrogen (Waltham, MA, United States). Coverslip sealant (CoverGrip™) was obtained from Biotium (Fremont, CA, United States). Blocking One Histo was obtained from Nacalai Tesque (Kyoto, Japan). Triton® X-100 and phosphate-buffered saline (PBS) were acquired from Thermo Scientific Chemicals (Waltham, MA, United States).

### 2.2 Methods

#### 2.2.1 Cell culture

Human BMECs (HBEC-5i; passages 21–28) were cultured in endothelial cell medium supplemented with 5% fetal bovine serum (FBS), 1% endothelial cell growth supplement (ECGS), and 1% penicillin–streptomycin (P/S). Cells were propagated in T-75 culture flasks pre-coated overnight at room temperature with type I rat tail collagen (10 μ g/cm^2^). Human primary astrocytes and primary vascular pericytes (passages 3–8) were maintained in their respective specialized media, each supplemented with 2% FBS, 1% corresponding cell-specific growth supplement, and 1% P/S. Prior to seeding, T-75 flasks were coated overnight with poly-L-lysine (PLL; 5 μ g/cm^2^).

All cells were incubated at 37 °C in a humidified atmosphere containing 5% ${\text{CO}}_{2}$ . Medium exchanges occurred every 2–3 days, and passaging was performed at 80%–90% confluency. Cell morphology and viability were routinely monitored, and only cultures exhibiting optimal growth characteristics were utilized for subsequent experiments.

#### 2.2.2 BBB triculture model establishment

**FIGURE 1:**
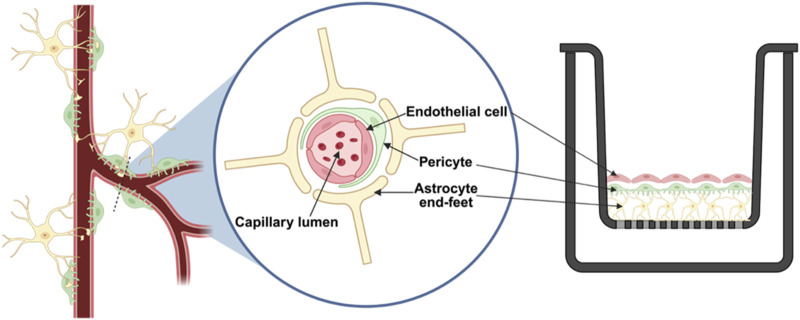
Schematic representation of the blood-brain barrier (BBB): Left, *in vivo* BBB structure; Middle, cross-sectional view of the BBB capillary; Right, our *in vitro* direct contact triculture Transwell® model, with astrocytes, pericytes, brain microvascular endothelial cells layered sequentially on the apical side of the insert to mimic the BBB.

Detailed cell culture conditions for the BBB were optimized utilizing a Design of Experiment (DOE) approach, as discussed in our previous publication (Lubin and Knipp, 2021). Briefly, the BBB model configuration is illustrated in Figure 1, where the *in vitro* direct contact triculture BBB model was established on Transwell® inserts (12 mm diameter, 0.4 μ m pore polyester membrane). Inserts were precoated overnight at room temperature with poly-L-lysine (PLL, 5 μ g/cm^2^) to promote cell adhesion. Human primary astrocytes were seeded onto the apical side of the inserts at 20,000 cells/cm^2^ and incubated for 48 h to form an initial adherent monolayer. Subsequently, human primary vascular pericytes were seeded directly onto the astrocyte layer at the same density (20,000 cells/cm^2^) and cultured for an additional 48 h to facilitate intercellular interactions. To mimic the extracellular matrix environment of the neurovascular unit, Matrigel® diluted in HBSS (5 μ L/cm^2^) was gently applied onto the astrocyte–pericyte bilayer and incubated at 37 °C for 30–45 min. Subsequently, Matrigel solution was carefully aspirated prior to seeding HBEC-5i at a density of 80,000 cells/cm^2^, completing the triculture assembly. Cultures were maintained in complete endothelial growth medium added to both apical and basolateral compartments, with medium exchanged every 48 h. The assembled BBB triculture was then incubated under standard conditions for 9 days post-endothelial cell seeding to allow for maturation of tight junctions, barrier integrity, and functional transporter expression before subsequent permeability assessments (Lubin and Knipp, 2021).

#### 2.2.3 BBB triculture model immunostaining

The BBB triculture model was contrasted with the simultaneous culture of HBEC-5i monocultures on the Transwell® membranes for 9 days to conduct immunofluorescence evaluation. On the 9th day after HBEC-5i cell seeding, the filter supports were rinsed with PBS and fixed with 4% paraformaldehyde (w/v in PBS) for 15 min at room temperature. Permeabilization was performed using 0.1% Triton® X-100 for 10 min, followed by blocking with Blocking One Histo for 15 min to minimize non-specific binding. To evaluate tight junction integrity and characteristics, cells were incubated with primary antibodies against occludin (FITC-conjugated, 15 μ g/mL), claudin-5 (Alexa Fluor™ 488-conjugated, 15 μ g/mL), ZO-1 (Alexa Fluor™ 488-conjugated, 5 μ g/mL). Efflux transporter expression was assessed using antibodies specific to P-gp (4 μ g/mL) and multidrug resistance–associated protein 2 (MRP2) (10 μ g/mL). All antibodies were diluted in 5% Blocking One Histo and incubated for 3 h at room temperature. For antibodies requiring secondary labeling, Alexa Fluor™ 488-conjugated goat anti-mouse IgG (0.5 μ g/mL) was applied for 45 min. Nuclei were counterstained with DAPI (5 μ g/mL) for 15 min. For cytoskeletal F-actin cytoskeletal visualization, fixed and permeabilized membranes were incubated with two drops/mL of ReadyProbes™ ActinRed™ 555 phalloidin conjugate (Invitrogen) in PBS for 30 min at room temperature. Following staining, membranes were carefully excised from the inserts, mounted on glass microscope slides using Permount™ mounting medium, and sealed with coverslips and a coverslip sealant. Imaging was conducted on a Nikon A1Rsi confocal microscope using a 60 × objective lens.

#### 2.2.4 Compound permeability assays

On day 9 post-endothelial (HBEC-5i) seeding, standardized bidirectional permeability assays were conducted using a panel of compounds as listed in Table 1. These compounds included CNS drugs, environmental toxicants, industrial chemicals, and emerging compounds of regulatory concern, at 50 μ M or 100 μ M in HBSS containing ≤ 1% DMSO. For apical-to-basolateral (A → B; blood-to-brain) transport, compounds were added to the apical (donor) chamber, and 200 μ L samples were collected from the basolateral (receiver) chamber at 30, 60, 90, 120, 150, and 180 min. At each time point, 200 μ L of fresh HBSS was replenished in the basolateral chamber to maintain volume equilibrium. For basolateral-to-apical (B → A; brain-to-blood) transport, the same protocol was applied with compounds introduced into the basolateral chamber and samples collected from the apical chamber. Each compound was tested in triplicate (n = 3). All samples were analyzed by high performance liquid chromatography (HPLC), as described in Supplementary Table S1. The $P_{\mathrm{app}}$ , in cm/sec , is calculated using the Equation 1:

$$P_{\mathrm{app}}=\frac{dM/dt}{\left[C_{0}\right]\cdot SA\cdot 60}$$ (1)

Where dM / dt represents the amount of compound (M) traversing the cell layer as a function of time (t) , $\left[C_{0}\right]$ is the initial concentration of compound in the donor chamber, SA is the surface area of the filter support, and 60 is a correction factor that converts from minutes to seconds.

### 2.3 Computational modeling

*In vitro* BBB permeability data were quantitatively extrapolated to estimate *in vivo* brain uptake kinetics and distribution. Specifically, we used the measured bidirectional $P_{\mathrm{app}}$ , for each compound to predict (1) the unidirectional brain uptake half-life under acute exposure, and (2) the steady-state brain concentration $\left(\left[C_{ss,brain}\right]\right)$ under chronic exposure. This approach integrated the *in vitro* BBB assay results with chemical-specific toxicokinetic parameters from the EPA’s HTTK-R package (Pearce et al., 2017; Wambaugh et al., 2024), including the fraction unbound in blood $\left(F_{ub}\right)$ and predicted steady-state serum concentration $\left(\left[C_{ss,serum}\right]\right)$ (Breen et al., 2021; Wambaugh et al., 2021).

#### 2.3.1 Acute exposure: Unidirectional brain uptake half-life

Brain uptake following an acute exposure was modeled as a first-order process from blood to the brain. We assumed an initial phase of unidirectional influx, during which brain levels are negligible relative to blood (sink conditions), so back-diffusion can be ignored (Chowdhury et al., 2021). Under these conditions, the rate of change in blood concentration due to BBB uptake is given by Equation 2:

$$\frac{dC}{dT}=-F_{ub}\cdot A_{\mathrm{BBB}}\cdot P_{\mathrm{app}}^{AB}\cdot \left[C\right]$$ (2)

where C is the mass of the compound, [C] is the chemical concentration in blood adjacent to the brain capillaries, $F_{ub}$ is the unbound fraction in blood, $A_{\mathrm{BBB}}$ is the total surface area of brain capillary endothelium, and $P_{\mathrm{app}}^{AB}$ is the apparent permeability (cm/s) from the *in vitro* BBB assay in the apical to basal direction. We assumed a standard $A_{\mathrm{BBB}}$ of 20 $m^{2}$ for an adult human brain, consistent with literature values for total brain microvascular surface area (Begley and Brightman, 2003). In this approach, we do not take into account ionization of acidic and basic compounds. The product $F_{ub}\cdot A_{\mathrm{BBB}}\cdot P_{\mathrm{app}}$ has units of volume per unit time and represents the permeability–surface area transport clearance for the compound. We defined the first-order uptake rate constant k (in units of 1/time ) in Equation 3:

$$k=F_{ub}\cdot A_{\mathrm{BBB}}\cdot P_{\mathrm{app}}^{AB}$$ (3)

which describes the fractional loss of compound from the blood compartment per unit of time due to BBB uptake. For additional details on this derivation, see Section 2.1 in the supplement. The corresponding half-life for brain uptake, $t_{1/2}$ , was calculated as $t_{1/2}=ln\left(2\right)/k$ . This $t_{1/2}$ represents the time required for the blood concentration (in the absence of other elimination routes) to decline by 50% as a result of net transfer into the brain. We used compound-specific $F_{ub}$ values (HTTK) in these calculations to account for plasma protein binding, since only the unbound fraction is available to diffuse across the BBB (Chowdhury et al., 2021).

#### 2.3.2 Chronic exposure: Steady-state brain concentrations

For chronic exposure scenarios, the steady-state concentration in brain tissue $\left(\left[C_{ss,brain}\right]\right)$ was estimated by integrating *in vitro* BBB permeability data with systemic exposure predictions. At steady state, the influx and efflux of a compound across the BBB equilibrate, establishing a constant ratio between the unbound concentrations in brain and plasma, which is governed by passive permeability and active transport processes (Storelli et al., 2021). For the steady state, we do not include the $F_{ub}$ , assuming that protein binding is similar in the blood and brain compartments. In addition, the ionization state of the compound is not considered. Although ionization can slow transfer between blood and tissues, under chronic exposure conditions this effect primarily increases the time required to reach steady state, particularly for compartments with pH values similar to that of blood. See the Supplementary Material, Section S2.2, for further justification. The relationship between the steady-state brain concentration $\left(\left[C_{ss,brain}\right]\right)$ and the serum steady-state concentration $\left(\left[C_{ss,serum}\right]\right)$ was defined as shown in Equation 4:

$$\left[C_{ss,brain}\right]=\left[C_{ss,serum}\right]\cdot \frac{P_{\mathrm{app}}^{AB}}{P_{\mathrm{app}}^{BA}}$$ (4)

This equation can also be expressed in terms of the efflux ratio (ER) as shown in Equation 5:

$$ER=\frac{P_{\mathrm{app}}^{BA}}{P_{\mathrm{app}}^{AB}}$$ (5)

Thus, brain concentration relationship is showing as Equation 6.

$$\left[C_{ss,brain}\right]=\frac{\left[C_{ss,serum}\right]}{ER}$$ (6)

Where, the ratio $P_{\mathrm{app}}^{AB}/P_{\mathrm{app}}^{BA}$ (*i.e.*, the reciprocal of ER) indicates the net proportion of compound at equilibrium in brain relative to plasma (Storelli et al., 2021). An ER value near 1 suggests no significant directional transport bias, resulting in brain concentrations approaching plasma concentrations at equilibrium $\left[C_{ss,brain}\right]\approx \left[C_{ss,serum}\right]$ . In contrast, a large ER (≥2.5) indicates strong apical efflux transporter activity, significantly reducing the equilibrium brain concentration relative to plasma (Varma et al., 2006; Seithel et al., 2006; Anderle et al., 2004). Conversely, low ER values (<0.4) suggest an active influx transport mechanism(s), a phenomenon often underappreciated due to prevailing emphasis on efflux transporters in the field.

Compound-specific steady-state serum concentrations, $\left[C_{ss,serum}\right]$ , were calculated using the HTTK-R (Pearce et al., 2017; Wambaugh et al., 2021; Wambaugh et al., 2024). HTTK-R employs generic PBTK models along with chemical-specific *in vitro* ADME data, including intrinsic clearance and $F_{ub}$ , to estimate systemic exposure concentrations under defined dosing regimens (e.g., 1mg/kg/day ) (Wambaugh et al., 2024). These predictions were linearly scaled to exposure scenarios relevant to environmental and toxicological risk assessments. Utilizing these simulated serum steady-state values, we calculated the corresponding steady-state brain concentrations $\left(\left[C_{ss,brain}\right]\right)$ for each compound as described above. See the Supplementary Tables S5, S6 for parameter values and HTTK-R calculated values.

Caco-2 data was obtained from the HTTK-R package (Pearce et al., 2017; R Core Team, 2021) release 2.3.0 (Wambaugh et al., 2024). This version of the HTTK-R package has Caco-2 permeability data for 11 of our compounds. See the Supplementary Table S4 for a summary of the Caco-2 values and our measured $P_{\mathrm{app}}^{AB}$ and $P_{\mathrm{app}}^{BA}$ values.

## 3 Results

### 3.1 BBB triculture model characterization with immunofluorescence staining

**FIGURE 2:**
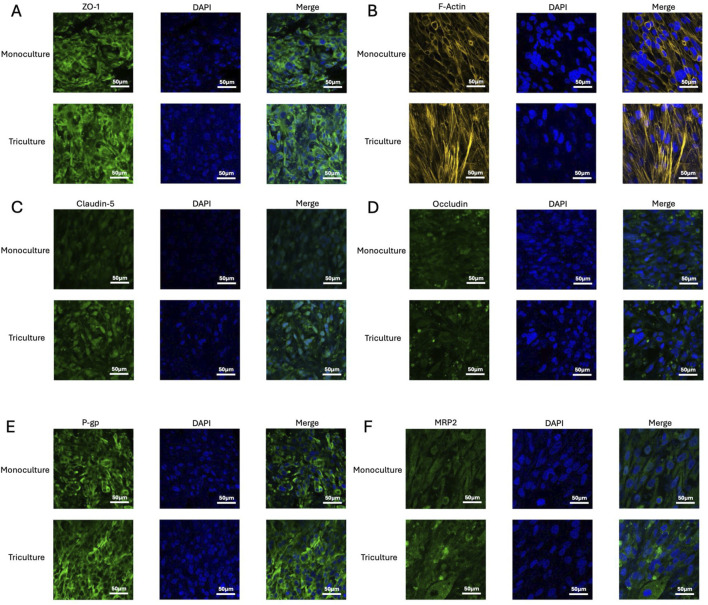
Immunofluorescence characterization of the *in vitro* BBB triculture model. Tight junction related proteins: **(A)** ZO-1, **(B)** F-Actin, **(C)** Claudin-5, and **(D)** Occludin. Efflux transporters: **(E)** P-gp and **(F)** MRP2 in the BBB triculture model and HBEC-5i monoculture controls. The triculture model exhibited stronger and more continuous junctional staining, enhanced efflux transporter localization, and a well-organized actin cytoskeleton compared with the weaker, discontinuous signals observed in monoculture. Scale bar = 50 μ m.

Immunofluorescence staining was employed to assess the barrier integrity and phenotypic characteristics of the developed *in vitro* direct-contact triculture BBB model comprised of human brain endothelial cells (HBEC-5i), primary astrocytes, and primary vascular pericytes. Key markers of tight junctions, cytoskeletal integrity, efflux transporter function, cytoskeletal integrity, and nuclear morphology were evaluated and compared with HBEC-5i monoculture controls (Figure 2).

In the triculture model, ZO-1 and F-actin exhibited stronger and more continuous expression compared with the HBEC-5i monoculture, reflecting enhanced cytoskeletal organization and tight junction integrity. The triculture configuration also displayed smaller intercellular gaps and more compact cellular alignment. Claudin-5 and occludin showed increased and continuous junctional localization, further supporting the establishment of a mature barrier phenotype.

Efflux transporters, including P-gp and MRP2, were expressed on the endothelial cell membrane under both conditions; however, the triculture model demonstrated higher and more polarized expression, consistent with upregulated transporter activity observed under physiologically relevant conditions.

Together, these findings support the successful reconstitution of a human-relevant BBB phenotype in the triculture model, with significantly enhanced junctional integrity, cytoskeletal organization, and efflux transporter expression relative to endothelial monocultures. This underscores the critical role of perivascular cell types in promoting and maintaining BBB-specific features *in vitro*.

### 3.2 BBB triculture model permeability data

To evaluate the barrier properties and functional transport dynamics of our *in vitro* BBB triculture system, we performed standardized bidirectional permeability assays on a chemically diverse panel of 49 compounds with varying CNS penetration profiles, toxicological significance, and pharmaceutical relevance. The results are shown in Table 1.

$P_{\mathrm{app}}$ was determined in both apical-to-basolateral (AB) and basolateral-to-apical (BA) directions. Compounds exhibited a wide range of permeability values, spanning three orders of magnitude. Low $P_{\mathrm{app}}^{AB}$ values (e.g., $<1\times 10^{-6}cm/s$ ) were observed for compounds such as DL-nicotine $\left(0.082\times 10^{-6}cm/s\right)$ , edaravone $\left(0.41\times 10^{-6}cm/s\right)$ and verapamil $\left(0.57\times 10^{-6}cm/s\right)$ , suggesting minimal passive diffusion. In contrast, highly permeable agents such as rifamycin SV $\left(45.9\times 10^{-6}cm/s\right)$ , methoxyphenol $\left(54.8\times 10^{-6}cm/s\right)$ , and paraquat $\left(21.9\times 10^{-6}cm/s\right)$ demonstrated rapid transfer across the endothelial barrier. In addition, two compounds, valproic acid and triadimefon, had very fast $P_{\mathrm{app}}^{AB}$ rates greater than $\geq 500\times 10^{-6}cm/s$ .

**FIGURE 3:**
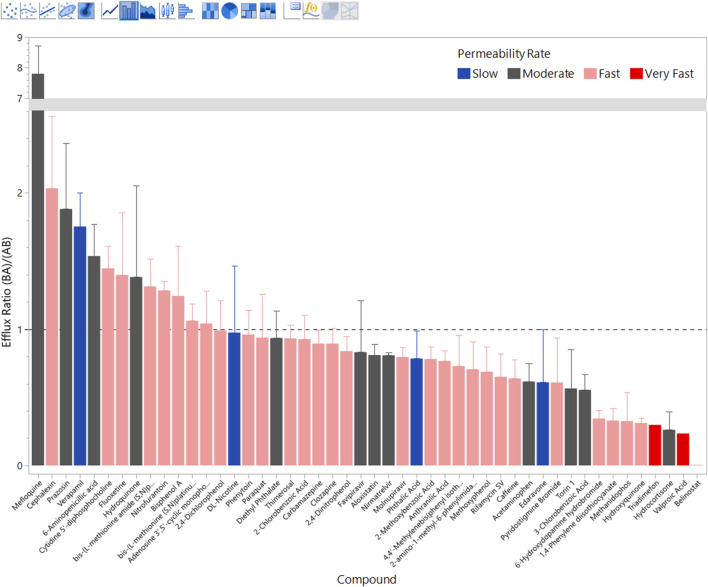
Calculated Efflux Ratios (ER). Efflux Ratios greater than one are active export into the simulated blood space, values less than one are active import. Bars are color coded by $P_{\mathrm{app}}^{AB}$ with red being fast and blue being slow. Error bars are estimated based on the largest error in the two $P_{\mathrm{app}}$ values. Belinostat, the smallest value, has an efflux ratio of 0.0037 ± 0.0044. Note the y-axis break between ER 3 and 7. The data is from Table 1.

ER , calculated as ( $P_{\mathrm{app}}^{AB}$ )/( $P_{\mathrm{app}}^{BA}$ ), provides insight into directional transport mechanisms. Two compounds, mefloquine (ER=7.8) , and cephalexin (*ER* = 2.03), exhibited notable asymmetry favoring basolateral-to-apical movement, indicative of active efflux transport. Conversely, compounds like hydrocortisone (*ER* = 0.26), hydroxyquinone (*ER* = 0.31), and belinostat (*ER* = 0.0038) showed preferential *Ap* → *Bl* movement, suggesting potential active uptake or reduced efflux. ER values are shown in Table 1 and Figure 3.

Notably, some clinically relevant neuroactive agents, including caffeine and acetaminophen, displayed relatively symmetric permeability (ER≈0.6) , aligning with their known ability to cross the BBB.

In summary, the bidirectional $P_{\mathrm{app}}$ spanned a broad dynamic range, with $P_{\mathrm{app}}^{AB}$ values ranging from $0.08\times 10^{-6}$ to $56.1\times 10^{-6}cm/s$ and $P_{\mathrm{app}}^{BA}$ values from $0.07\times 10^{-6}$ to $149\times 10^{-6}cm/s$ , covering three orders of magnitude. Notably, eight compounds exhibited permeability that was either too rapid or confounded by metabolic instability to permit reliable $P_{\mathrm{app}}$ determination, and four compounds exhibited limited permeability due to compound properties or limited solubility, these compounds are listed in Supplementary Tables S2, S3.

These findings demonstrate the capability of our triculture model to discriminate between compounds with diverse permeability profiles and transporter interactions, supporting its utility for neuropharmacokinetic screening and IVIVE modeling of brain exposure.

### 3.3 Acute uptake half-life estimation

To characterize the kinetics of unbound drug entry into the brain following a single exposure, we estimated unidirectional uptake half-lives $\left(t_{1/2}\right)$ (Equation 2) for each compound using experimentally derived $P_{\mathrm{app}}^{AB}$ along with $F_{ub}$ sourced from the HTTK-R database. These values were applied to a physiologically relevant scale assuming a brain capillary surface area of $20m^{2}$ in humans (Kaya and Ahishali, 2021).

**FIGURE 4:**
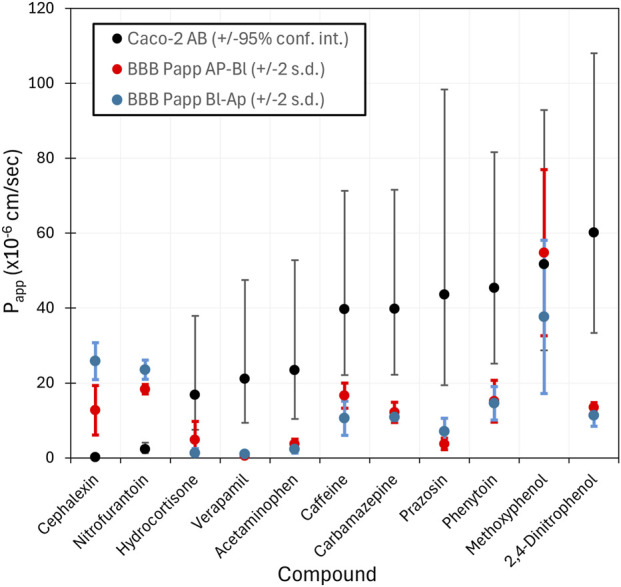
Comparison of Caco-2 ${\text{P}}_{\mathrm{app}}$ (black) with our BBB $P_{\mathrm{app}}^{AB}$ values (red) and $P_{\mathrm{app}}^{BA}$ values (blue). Caco-2 values are from the HTTK-R toolkit. The compounds are sorted by increasing Caco-2 ${\text{P}}_{\mathrm{app}}$ . Error bars are ± 95% confidence limits on the Caco-2 data and two standard deviations for our $P_{\mathrm{app}}^{AB}$ values. The correlation between Caco-2 (AB) with our $P_{\mathrm{app}}^{AB}$ is 0.11. This data is given in Supplementary Table S4.

Estimated uptake half-lives, shown in Figure 4, spanned a wide range, reflecting compound-specific permeability and binding characteristics. Highly permeable, low-binding compounds, such as CNS-active drugs, exhibited rapid brain uptake kinetics, with uptake rate constants (*k*) on the order of 0.1/*min* and corresponding half-lives of 1–10 min. In contrast, poorly permeable or highly protein-bound chemicals displayed slower kinetics, with rate constants closer to 0.005/min, yielding estimated *t*_1/2_ values of 1–10 h.

For instance, belinostat, a CNS-penetrating histone deacetylase inhibitor with high $P_{\mathrm{app}}^{AB}$ and negligible efflux, was predicted to reach equilibrium in the brain within minutes $\left(t_{1/2}\approx 5\;min\right)$ . Conversely, a hydrophilic compound, such as phthalic acid, exhibited much slower predicted brain uptake $\left(t_{1/2}>2\;hours\right)$ , consistent with its low permeability.

Overall, compounds with $P_{\mathrm{app}}$ values exceeding $∼3\times 10^{-6}cm/s$ demonstrated favorable brain penetration potential, while those below $∼1\times 10^{-6}cm/s$ showed limited access to the CNS under acute exposure conditions. These findings reinforce the utility of incorporating both *in vitro* permeability and plasma protein binding data to quantitatively estimate the early brain exposure dynamics of xenobiotics. A full summary of calculated half-lives is provided in Figure 4 and in tables in the Supplementary Material.

### 3.4 Estimated steady-state brain concentrations

**FIGURE 5:**
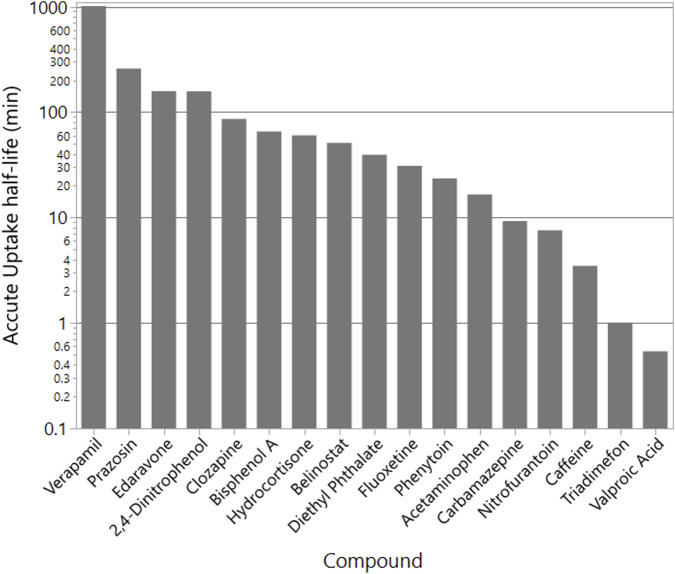
Estimated initial uptake half-lives of compounds reported to have either beneficial or toxic neurological effects. Compounds are sorted from slowest to fastest uptake half-lives. This data is given in Supplementary Table S5.

We estimated steady-state brain concentrations $\left(\left[C_{ss,brain}\right]\right)$ by integrating experimentally determined bidirectional permeability coefficients with HTTK-predicted plasma concentrations. The results revealed substantial variability in brain exposure across the tested compounds, driven primarily by differences in BBB efflux ratio as shown in Figure 5.

Compounds with high permeability and minimal efflux, such as belinostat, were predicted to reach brain concentrations much higher than their plasma concentrations. Belinostat exhibited a remarkably low efflux ratio (∼0.004) , resulting in efficient brain accumulation. Other compounds predicted to accumulate in the brain included hydrocortisone, triadimefon, edaravone, caffeine and acetaminophen.

In contrast, compounds with high efflux ratios (e.g., mefloquine and 6-hydroxydopamine) showed marked attenuation in predicted brain levels despite moderate to high systemic exposure. For example, 6-hydroxydopamine displayed an efflux ratio > 5, leading to a significant reduction in brain partitioning. Such trends highlight the functional impact of active efflux in limiting CNS exposure.

Across the dataset, compounds with efflux ratios ≥2.5 typically showed $\left[C_{ss,brain}\right]$ values below 50% of the unbound plasma concentration, while those with ratios <0.4 tended to accumulate in the brain at higher levels. These results align with known *in vivo* data for several CNS-active and neurotoxic compounds, supporting the predictive utility of our integrated BBB-HTTK framework. A summary of compound-specific predicted $\left[C_{ss,brain}\right]$ values and associated parameters is provided in Supplementary Tables S4, S5.

Using Equation 2 and our measured efflux ratios, we estimated the steady state brain concentrations shown in Figure 5. Comparing the HTTK-R $\left[C_{ss,serum}\right]$ values to the $\left[C_{ss,brain}\right]$ values we calculated based on our measured permeability values suggest significant change in steady state concentrations in the brain emerge as the permeability changes. In general, the calculated [*C_ss,brain_*] concentrations are lower than the calculated serum values. This is consistent with our understanding that the BBB is more restrictive in the passage of compounds and suggests that our *in vitro* assay reflects this increased barrier function. Some compounds, like verapamil, are predicted to have only 1%–2% the serum concentration in the brain which is ten fold lower than in the serum. In contrast, belinostat is predicted to have a concentration more than 100 times higher in the brain compared to serum.

## 4 Discussion

### 4.1 *In Vitro* model performance and validation

The human *in vitro* direct-contact triculture BBB model established in this study demonstrated physiologically relevant barrier properties, closely mimicking the *in vivo* neurovascular interface. Compared with monoculture conditions, the inclusion of astrocytes and pericytes enhanced tight junction formation, as evidenced by robust immunofluorescent staining of ZO-1, F-actin, occludin, and claudin-5. Efflux transporters P-gp and MRP2 also showed increased expression, confirming the functional phenotype of the triculture system. The model maintained stable morphology and consistent permeability behavior over time, supporting its suitability for screening CNS permeability and assessing neurotoxicity potential.

### 4.2 Permeability profiling and directionality

Our measured bi-directional apparent permeability coefficients ( $P_{\mathrm{app}}^{AB}$ ) covered over three orders of magnitude, ranging from highly restrictive compounds (e.g., DL-nicotine, $P_{\mathrm{app}}^{AB}\approx 0.08\times 10^{-6}cm/s$ ) to freely diffusing agents (e.g., rifamycin SV, $P_{\mathrm{app}}>46\times 10^{-6}cm/s$ ). The low $P_{\mathrm{app}}^{AB}$ measured for DL-nicotine reflects its predominantly protonated state under physiological conditions, which limits passive diffusion across a tight BBB. Higher nicotine permeability values reported under different experimental conditions reflect transport of free-base nicotine and differences in barrier restrictiveness (Garberg et al., 2005; Jorgensen et al., 2025). Directional transport behavior was characterized by ERs, which provided insight into the influence of active transporters (see Figure 1). Several known P-gp substrates, such as mefloquine, cephalexin, and prazosin, exhibited *ER* >1.8, suggesting significant efflux limitation on net brain entry. Conversely, compounds with *ER* <1, including belinostat and hydrocortisone, indicated net influx or minimal efflux activity. Consistent with the CNS disposition framework described in prior work, our triculture $P_{\mathrm{app}}^{AB}$ values for carbamazepine, clozapine, fluoxetine, and phenytoin are in the same order of magnitude (reported 30.9, 28.3, 6.4, and $27.2\times 10^{-6}cm/s$ vs. our 12.2, 13.4, 14.4, and $15.1\times 10^{-6}cm/s$ ; ER 0.89–1.4 ) (Summerfield et al., 2007). The ∼2–3 -fold lower $P_{\mathrm{app}}^{AB}$ for carbamazepine, clozapine, and phenytoin is plausible given the literature used an MDCK monolayer assay (Summerfield et al., 2007). *In vivo* $f_{u,brain}$ spans nearly two orders of magnitude across these compounds (carbamazepine 0.1185; clozapine 0.0110; fluoxetine 0.0040; phenytoin 0.0082), indicating that similar BBB permeability can still yield different unbound brain exposure due to differences in brain tissue binding. The ER data is shown in Figure 3.

### 4.3 Comparison with Caco-2 and BBB triculture model data

We extracted Caco-2 $P_{\mathrm{app}}$ data from HTTK-R (Pearce et al., 2017), version 2.3.1 (Wambaugh et al., 2024). This version of HTTK-R has measured Caco-2 permeability data (Honda et al., 2024) for 11 of our compounds. The Caco-2 assay is an *in vitro* test using human epithelial colorectal adenocarcinoma cells—that differentiate to form a monolayer resembling the intestinal lining (Lee et al., 2017). It is widely used to evaluate intestinal drug absorption and permeability in pharmaceutical and toxicological research (Honda et al., 2024).

**FIGURE 6:**
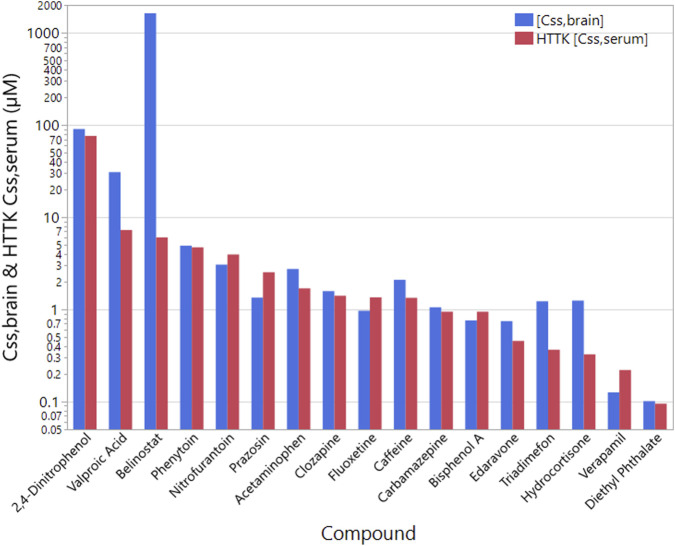
Estimated steady state brain concentrations for chronic exposure. Values are μM . Compounds are ordered by decreasing $\left[C_{ss,serum}\right]$ . Blue bars are the calculated $\left[C_{ss,brain}\right]$ and red columns the HTTK-R $\left[C_{ss,serum}\right]$ values. This data is given in Supplementary Table S5.

Figure 6 compares the Caco-2 HTTK values versus our measured $P_{\mathrm{app}}^{AB}$ values. In general, the BBB triculture measured permeabilities are lower than the Caco-2 permeabilities especially when the later is large. Exceptions to this trend are the notably higher BBB triculture values for cephalexin, and nitrofurantoin. In a direct comparison, the correlation R-squared between Caco-2 (AB) and our $P_{\mathrm{app}}^{AB}$ is 0.11.

### 4.4 Acute brain uptake kinetics

Using HTTK-R unbound fraction data $\left(F_{ub}\right)$ and our measured A → B permeability values, we estimated unidirectional brain uptake half-lives $\left(t_{1/2}\right)$ under an acute exposure scenarios (Figure 4). These ranged from less than 1 h for high-permeability, compounds to 100–1,000 h for poorly permeable or actively exported compounds. For example, triadimefon was predicted to reach half-maximal brain accumulation in 60 min. Whereas verapamil and prazosin, both P-gp substrates, are estimated to require more than 100 h.

These predictions aligned with established thresholds, where compounds with $P_{\mathrm{app}}>3\times 10^{-6}cm/s$ generally exhibited rapid CNS penetration, while those below $∼1\times 10^{-6}cm/s$ accumulated slowly or minimally in the brain (Wang et al., 2005).

### 4.5 Steady-state brain concentration estimation

To extend our findings to chronic exposure contexts, we combined *P_app_* and efflux ratios with data from HTTK-R to estimate unbound steady-state brain concentrations [*C_ss,brain_*] (Figure 5). By scaling [*C_ss,serum_*] using our ER derived values, we obtained compound-specific brain distribution predictions. In particular, compounds with high systemic exposure but significant efflux activity, such as prazosin and verapamil, exhibited markedly lower brain concentrations than their plasma levels. In contrast, CNS-targeting agents with minimal efflux (e.g., acetaminophen, hydrocortisone and triadimefon) reach higher concentrations in brain versus plasma. These estimates provide a critical link between *in vitro* barrier transport and expected *in vivo* brain exposure.

### 4.6 Implications for neurotoxicity and drug screening

The integration of permeability screening with HTTK modeling offers a powerful framework for prioritizing neuroactive or neurotoxic compounds in early-stage development or regulatory evaluation. Our model captures both the physical barrier function and directional transport mechanisms of the human BBB, allowing for a more predictive assessment of brain exposure potential. The ability to estimate acute uptake kinetics and steady-state levels using experimentally derived parameters enhances the translational value of *in vitro* testing, particularly for environmental or industrial chemicals lacking robust pharmacokinetic data.

### 4.7 Limitations and future directions

Although the triculture model captures key features of the neurovascular unit, several limitations remain. The HBEC-5i cell line, though widely used, may not fully replicate the transporter profile and functional heterogeneity of primary human brain microvascular endothelial cells. In addition, immune-responsive components such as microglia are currently not included, limiting the applicability of the model to non-neuroinflammatory conditions. Future work will focus on integrating immune cells and evaluating inflammatory barrier responses. Moreover, expanding validation against *in vivo* brain/plasma ratios will strengthen the confidence in IVIVE predictions derived from this platform.

## 5 Conclusion

In summary, our BBB triculture model, coupled with toxicokinetic modeling, enables predictions of brain exposure across a chemically diverse set of compounds. This model allows better estimates of xenobiotic concentrations in the brain and may facilitate IVIVE in pharmacological and toxicological testing. Thus offering an alternative to animal testing in line with evolving regulatory frameworks and the goals of next-generation risk assessment.

## Data Availability

The original contributions presented in the study are included in the article/Supplementary Material, further inquiries can be directed to the corresponding authors.
