# Correction: *In Vitro* BBB triculture assay and preliminary computational model development to predict brain exposure

**DOI:** 10.3389/ftox.2026.1836636

**Published:** 2026-04-07

**Authors:** Zhuangyan Monica Xu, James P. Sluka, Charlie C. Zhang, Gregory Knipp

**Affiliations:** 1 Department of Industrial and Molecular Pharmaceutics, College of Pharmacy, Purdue University, West Lafayette, IN, United States; 2 Biocomplexity Institute, Intelligent Systems Engineering, Indiana University, Bloomington, IN, United States

**Keywords:** apparent permeability, blood-brain barrier, CNS exposure prediction, efflux ratio, high throughput toxicokinetics in R, HTTK-R, in vitro-in vivo, IVIVE

In the originally published article, the text did not indicate the corresponding data sources (**Table 1** and **Figure 3**) for the reported ER values. A sentence has been added to clarify this point as “ER values are shown in **Table 1** and **Figure 3**.”

A correction has been made to the section **3 Results**, *3.2 BBB triculture model permeability data*, Paragraph Number of 03:

“ER, calculated as ( $P_{app}^{AB}/P_{app}^{BA}$ ), provides insight into directional transport mechanisms. Two compounds, mefloquine ( ER = 7.8), and cephalexin ( ER = 2.03), exhibited notable asymmetry favoring basolateral-to-apical movement, indicative of active efflux transport. Conversely, compounds like hydrocortisone ( ER = 0.26), hydroxyquinone ( ER = 0.31), and belinostat ( ER = 0.0038) showed preferential Ap → Bl movement, suggesting potential active uptake or reduced efflux. ER values are shown in **Table 1** and **Figure 3**.”

The figure numbering in the section **3 Results**, *3.3 Acute uptake half-life estimation,* Paragraph Number of 02 is incorrect. The text previously stated, “Estimated uptake half-lives, shown in **Figure 3**,” and has been corrected to “Estimated uptake half-lives, shown in **Figure 4**”.

A correction has been made to the section **3 Results**, *3.3 Acute uptake half-life estimation,* Paragraph Number of 02:

“Estimated uptake half-lives, shown in **Figure 4**, spanned a wide range, reflecting compound-specific permeability and binding characteristics. Highly permeable, low-binding compounds, such as CNS-active drugs, exhibited rapid brain uptake kinetics, with uptake rate constants ( k ) on the order of 0.1 /⁡min and corresponding half-lives of 1–10 min. In contrast, poorly permeable or highly protein-bound chemicals displayed slower kinetics, with rate constants closer to 0.005/min, yielding estimated $t_{1/2}$ values of 1–10 h.”

The figure numbering in the section **3 Results**, *3.3 Acute uptake half-life estimation,* Paragraph Number of 04 is incorrect. The text previously stated, “A full summary of calculated half-lives is provided in **Figure 3** and in tables in the **Supplementary Material**.” The corrected text appears below: “A full summary of calculated half-lives is provided in **Figure 4** and in tables in the **Supplementary Material**.”

A correction has been made to the section **3 Results**, *3.3 Acute uptake half-life estimation,* Paragraph Number of 04:

“Overall, compounds with $P_{app}$ values exceeding ∼3 × 10^−6^ cm/s demonstrated favorable brain penetration potential, while those below ∼1 × 10^−6^ cm/s showed limited access to the CNS under acute exposure conditions. These findings reinforce the utility of incorporating both *in vitro* permeability and plasma protein binding data to quantitatively estimate the early brain exposure dynamics of xenobiotics. A full summary of calculated half-lives is provided in **Figure 4** and in tables in the **Supplementary Material**.”

The figure numbering in the section **3 Results**, *3.4 Estimated steady-state brain concentrations,* Paragraph Number of 01 is incorrect. The text previously stated, “we estimated the steady state brain concentrations shown in **Figure 4**”, and should be corrected to “we estimated the steady state brain concentrations shown in **Figure 5**.”

A correction has been made to the section **3 Results**, *3.4 Estimated steady-state brain concentrations,* Paragraph Number of 01:

“We estimated steady-state brain concentrations ([ $C_{ss,brain}$ ]) by integrating experimentally determined bidirectional permeability coefficients with HTTK-predicted plasma concentrations. The results revealed substantial variability in brain exposure across the tested compounds, driven primarily by differences in BBB efflux ratio as shown in **Figure 5**.”

The figure numbering in the section **3 Results**, *3.4 Estimated steady-state brain concentrations*, Paragraph Number of 05 is incorrect. The text previously stated, “we estimated the steady-state brain concentrations shown in **Figure 4**”, and should be corrected to “we estimated the steady-state brain concentrations shown in **Figure 5**.”

A correction has been made to the section **3 Results**, *3.4 Estimated steady-state brain concentrations*, Paragraph Number of 05:

“Using **Equation 2** and our measured efflux ratios, we estimated the steady state brain concentrations shown in **Figure 5**. Comparing the HTTK-R [ $C_{ss,serum}$ ] values to the [ $C_{ss,brain}$ ] values we calculated based on our measured permeability values suggest significant change in steady state concentrations in the brain emerge as the permeability changes. In general, the calculated [ $C_{ss,brain}$ ] concentrations are lower than the calculated serum values. This is consistent with our understanding that the BBB is more restrictive in the passage of compounds and suggests that our *in vitro* assay reflects this increased barrier function. Some compounds, like verapamil, are predicted to have only 1%–2% the serum concentration in the brain which is ten fold lower than in the serum. In contrast, belinostat is predicted to have a concentration more than 100 times higher in the brain compared to serum.”

The figure numbering in the section **4 Discussion**, *4.2 Permeability profiling and directionality*, Paragraph Number of 01 is incorrect. The last sentence previously stated, “The ER data is shown in **Figure 5**.” and should be corrected to “The ER data is shown in **Figure 3**.”

A correction has been made to the section **4 Discussion**, *4.2 Permeability profiling and directionality*, Paragraph Number of 01:

“Our measured bi-directional apparent permeability coefficients ( $P_{app}^{AB}$ ) covered over three orders of magnitude, ranging from highly restrictive compounds (e.g., DL-nicotine, $P_{app}^{AB}$ ≈ 0.08 × 10^−6^ cm/s ) to freely diffusing agents (e.g., rifamycin SV, $P_{app}$ >46 × 10^−6^ cm/s ). The low $P_{app}^{AB}$ measured for DL-nicotine reflects its predominantly protonated state under physiological conditions, which limits passive diffusion across a tight BBB. Higher nicotine permeability values reported under different experimental conditions reflect transport of free-base nicotine and differences in barrier restrictiveness (**Garberg et al., 2005**; **Jorgensen et al., 2025**). Directional transport behavior was characterized by ERs, which provided insight into the influence of active transporters (see **Figure 1**). Several known P-gp substrates, such as mefloquine, cephalexin, and prazosin, exhibited ER >1.8, suggesting significant efflux limitation on net brain entry. Conversely, compounds with ER <1, including belinostat and hydrocortisone, indicated net influx or minimal efflux activity. Consistent with the CNS disposition framework described in prior work, our triculture $P_{app}^{AB}$ values for carbamazepine, clozapine, fluoxetine, and phenytoin are in the same order of magnitude (reported 30.9, 28.3, 6.4, and 27.2 × 10^−6^ cm/s vs. our 12.2, 13.4, 14.4, and 15.1 × 10^−6^ cm/s ; ER 0.89–1.4) (**Summerfield et al., 2007**). The ∼ 2–3-fold lower $P_{app}^{AB}$ for carbamazepine, clozapine, and phenytoin is plausible given the literature used an MDCK monolayer assay (**Summerfield et al., 2007**). *In vivo* $f_{u,brain}$ spans nearly two orders of magnitude across these compounds (carbamazepine 0.1185; clozapine 0.0110; fluoxetine 0.0040; phenytoin 0.0082), indicating that similar BBB permeability can still yield different unbound brain exposure due to differences in brain tissue binding. The ER data is shown in **Figure 3**.”

In the originally published article, the sentence “Using HTTK-R unbound fraction data ( $F_{ub}$ ) and our measured A→ B permeability values, we estimated unidirectional brain uptake half-lives ( $t_{1/2}$ ) under an acute exposure scenarios.” did not indicate the corresponding data source. The corrected sentence appears below: “Using HTTK-R unbound fraction data ( $F_{ub}$ ) and our measured A→ B permeability values, we estimated unidirectional brain uptake half-lives ( $t_{1/2}$ ) under an acute exposure scenarios (**Figure 4**).”

A correction has been made to the section **4 Discussion**, *4.4 Acute brain uptake kinetics*, Paragraph Number of 01:

“Using HTTK-R unbound fraction data ( $F_{ub}$ ) and our measured A→ B permeability values, we estimated unidirectional brain uptake half-lives ( $t_{1/2}$ ) under an acute exposure scenarios (**Figure 4**). These ranged from less than 1 h for high-permeability, compounds to 100–1,000 h for poorly permeable or actively exported compounds. For example, triadimefon was predicted to reach half-maximal brain accumulation in 60 min. Whereas verapamil and prazosin, both P-gp substrates, are estimated to require more than 100 h.”

In the originally published article, the sentence “To extend our findings to chronic exposure contexts, we combined $P_{app}$ and efflux ratios with data from HTTK-R to estimate unbound steady-state brain concentrations [ $C_{ss,brain}$ ].” did not indicate the corresponding data sources. The corrected sentence appears below: To extend our findings to chronic exposure contexts, we combined $P_{app}$ and efflux ratios with data from HTTK-R to estimate unbound steady-state brain concentrations [ $C_{ss,brain}$ ] (**Figure 5**).”

A correction has been made to the section **4 Discussion**, *4.5 Steady-state brain concentration estimation,* Paragraph Number of 01:

“To extend our findings to chronic exposure contexts, we combined $P_{app}$ and efflux ratios with data from HTTK-R to estimate unbound steady-state brain concentrations [ $C_{ss,brain}$ ] (**Figure 5**). By scaling [ $C_{ss,serum}$ ] using our ER derived values, we obtained compound-specific brain distribution predictions. In particular, compounds with high systemic exposure but significant efflux activity, such as prazosin and verapamil, exhibited markedly lower brain concentrations than their plasma levels. In contrast, CNS-targeting agents with minimal efflux (e.g., acetaminophen, hydrocortisone and triadimefon) reach higher concentrations in brain versus plasma. These estimates provide a critical link between *in vitro* barrier transport and expected *in vivo* brain exposure.”

The original article has been updated.

