# Supplementary material for: In Vitro BBB triculture assay and preliminary computational model development to predict brain exposure
Source: Front Toxicol. 2026 Mar 10;8:1781588. doi: 10.3389/ftox.2026.1781588 (PMC13008315; doi:10.3389/ftox.2026.1781588)
Supplement: Supplementary file 1 [file DataSheet1.pdf]

Supplementary material for:
In Vitro BBB Triculture Assay and Preliminary Computational Model Development to Predict Brain Exposure

February 9, 2026

1 Compounds Studied

1.1 Analytical Methods and Compounds Studied

The analytical details and CAS registry number (**CASRN**), logP, water solubility, molecular weight (MW), and pKa, for the compounds studied are given in **Table S1**.

Two compounds studied do not have CASRN numbers and are shown in **Figure S1**.

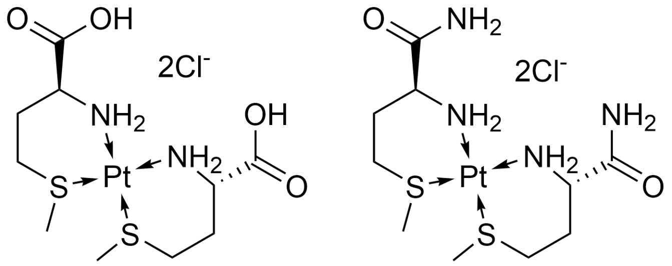

Figure S1: Compounds without CAS registry numbers. Left: bis-(L-methionine)-(S,N)platinum(II) dichloride (**BisMetPt**), Right: bis-(L-S-methionine amide)-(S,N)platinum(II) dichloride (**BisMetPtNH2**). See Behymer, et al. (2024), Toxicological Sciences 197(2), 197–210. `https://academic.oup.com/toxsci/article/197/2/197/741318`.

Table S1: Analytical Methods

Chemical	CASRN	HPLC Method	Flow Rate (mL/min)	Absoption Wave-length (nm)	Run time (min)	logP	Solubility (water, unless specified)	MW	pKa
Acetaminophen	103-90-2	20% Methanol:80% Water:0.1% TFA	1	250	10	0.46	14mg/mL 25C	151.16	9.38
Acrolein	107-02-8	5% Methanol:95% Water:0.1% TFA	0.5	210	10	-0.01	100mg/mL 70F	56.06	
Acrolein Diethylacetal	3054-95-3	5% Methanol:95% Water:0.1% TFA	0.5	224	15	1.4	soluble	130.18	
Acrylamide	79-06-1	5% ACN:95% Ammonium Formate (10mM)	0.35	210		-0.67	390mg/mL 25C	71.08	
Aloxistatin	88321-09-9	95% Methanol:5% Water:0.1% TFA	1	205	20			342.44	
2-Amino-1-methyl-6-phenylimidazo[4,5-b]-pyridine	105650-23-5	70% ACN:30% Water	1	316	13	2.23	soluble in DMSO	224.26	5.4
6-Aminopenicillic acid	551-16-6	3% ACN:97% Ammonium Formate (10mM)	0.5	240	18	-2.1		216.26	2.3
Amiodarone	1951-25-3	50% ACN:50% Ammonium Formate (10mM):0.1% Formic Acid	1	242	21			645.32	
Anthranilic acid	118-92-3	5% ACN:95% Water:0.1% TFA	1	247	18	1.21	3.5mg/mL	137.14	2.14
Atazanavir	198904-31-3	50% ACN:50% Water:0.1% Acetic acid	1	250				704.87	
Azodicarbonamide	123-77-3	90% ACN:10% Water:0.1% Formic acid	0.8	242	18			116.08	
Belinostat	414864-00-9	40% Methanol:60 % Water:0.1% TFA	1	266	17	1.7	0.14mg/mL	318.3	7.87
Benzyl isothiocyanate	622-78-6	60% Methanol:40% Water:0.1% TFA	1	342	14				
Bis(2-ethylhexyl) phthalate	117-81-7	95% Methanol:5% Water:0.1% TFA	1	230	12	7.6	0.27mg/L	390.6	
Bis(2-methoxyethyl) phthalate	117-82-8	70% Methanol:30% Water:0.1% TFA	1	225	8	1.4	8.5mg/mL	282.29	
Bisphenol A	80-05-7	3% Methanol:97% Water:0.1% TFA	2	224	15	3.32	0.3mg/mL	228.29	9.6
Bromoxynil	1689-84-5	50% ACN:50% Formate (0.1mM)	0.5	221	10			276.92	
Bupropion	34911-55-2	60% Acetonitrile:10% Water:0.1% TFA	1	254	20	3.6	312mg/mL	239.74	7.9
Caffeine	58-08-2	40% Methanol:60 % Water	1	272	11	-0.07	21.6mg/mL	194.19	14
Carbamazepine	298-46-4	60% ACN:40% Formate (0.1mM):0.1% TFA	0.75	210	9	2.77	35.4ug/mL	236.27	13.9
Carvedilol	72956-09-3	50% ACN:50% Water:0.1% Acetic acid	1	230	6			406.48	
Cephalexin	15686-71-2	50% ACN:50% Formate (10 mM)	1	260	16	0.65	10mg/mL	347.4	5.2
2-Chlorobenzoic acid	118-91-2	20% ACN:5% Methanol:75% Water:0.1% TFA	0.5	230	12	2.05	2.09mg/mL	156.56	2.89
3-Chlorobenzoic acid	535-80-8	90% ACN:10% Water:0.1% Phosphate acid	1	230	11	2.68	450mg/L	156.56	3.82
Chlorpyrifos	2921-88-2	5% ACN:95% Water	0.5	225	15			350.57	
Cisplatin	15663-27-1	30% Methanol:30% ACN:40% Water	0.5	210	18			300.05	
Clozapine	5786-21-0	65% KH2PO4 (0.5M):30% ACN:5% Water:0.1% Formic acid	1	215	18	3.23	11.8mg/L	326.8	7.5
2,4-Dichlorophenol	120-83-2	60% Methanol:40% Water:0.1% TFA	1	220	20	3.06	5.55mg/mL	163.00	7.89
Diethyl phthalate	84-66-2	70% Methanol:30% Water:0.1% TFA	1	225	10			222.24	
2,4-Dinitrophenol	51-28-5	55% ACN:45% Water:0.1% TFA	1	260	8	1.67	27.6ug/mL	184.11	4.09
Diphyllin compound (DP3C01)		70% Methanol:30% Water:0.1% TFA	1	263	21				
Favipiravir	259793-96-9	5% Methanol:8% ACN:12% PBS (pH 4.5):75% Water:0.1% TFA	1	230	10	-0.6	slightly soluble	157.1	5.1
Fenofibrate	49562-28-9	50% Acetonitrile:50% Water:0.1% TFA	1	240	12	5.2	0.707mg/L	360.8	

Fluoxetine	54910-89-3	50% Acetonitrile:50% Water:10mM Formate	1	226	16	4.05	1.7mg/L	309.33	
Hydrocortisone	50-23-7	20% ACN:30% Methanol:50% Water:0.1% TFA	1	254	10	1.61	0.32mg/mL	362.5	
6-Hydroxydopamine hydrobromide	636-00-0	5% ACN:95% Water:0.1% TFA-gradiant	1	275	20			250.09	
Hydroxyquinone	2474-72-8	30% ACN:70% Formate (10mM pH 3.2) isocratic	1	220	14				
Justicdin A		80% Methanol:20% Water:0.1% TFA	1	220	15				
Methoxychlor	72-43-5	5% Methanol:95% Water	0.5	210	10	5.08	<1mg/mL	345.6	
Mefloquine	53230-10-7	45% ACN:55% Formate (10mM pH 3.2): 0.05% Formic acid	1	222	14	3.9	very slightly sol	378.31	8.6
Methamidophos	10265-92-6	25% ACN:25% Water:50% Formate (10mM):0.1% TFA	0.5	205	15			141.1	
Methoxyacetic Acid	625-45-6	5% Acetonitrile:95% Water:10mM Formate	0.4	210	9	-0.3	100mg/mL	90.08	3.57
2-Methoxybenzoic Acid	579-75-9	25% ACN:75% Water:0.1% TFA	1	215	12	1.59	5mg/mL 30°C	152.15	3.9
4-Methoxyphenol	150-76-5	40% ACN:60% Formate (10mM):0.1% TFA	1	200	15			124.14	
Metoclopramide HCl	7232-21-5	70% ACN:30% Water:0.1% TFA	1	250				336.26	
Molnupiravir	2349386-89-4	15% ACN:85% Water:0.1% TFA	0.5	235	20			329.31	
Nitrofurantoin	67-20-9	30% Acetonitrile:70% Water:10mM Formate	1	370	16	-0.47	35.7ug/mL	238.16	7.2
DL-Nicotine	22083-74-5	40% ACN:60% Ammonia Formate (10mM)	1	220	11	1.2		162.23	
Nirmatrelvir	2628280-40-8	95% Methanol:5% Water:0.1% TFA	0.3	215	9	2.2		499.5	
Paraquat	4685-14-7	10% ACN:90% Phosphate (pH 2.5)	0.5	220	15	-4.22	620mg/mL	186.25	
1,4-Phenylene disothiocyanate	4044-65-9	20% Acetonitrile: 80% Water:10mM Formate	1	254	20	4.7	practically insol	192.3	
Phenytoin	57-41-0	40% ACN:60% Water:0.1% TFA	1	220	20	2.47	37.8ug/mL	252.27	8.33
Phthalic Acid	88-99-3	30% Methanol:70% Water:0.1% TFA	1	254	15	0.73	6.96mg/mL	166.13	2.76
Prazosin	19216-56-9	25% Acetonitrile: 75% Water:10mM Formate	1	246	15	1.3	1.4mg/mL	383.4	6.54
Pramiperoxole 2HCl	104632-25-9	70% ACN:30% Water:0.1% TFA	1	250	13			284.25	
Pyridostigmine Bromide	101-26-8	50% ACN:50% Formate (10 mM pH 3.2)	0.6	215	30		freely sol	261.12	
Ritonavir	155213-67-5	80% ACN:20% Formate (10 mM)	1	254	25	3.9	$1.1x10^{-4}$mg/L	720.9	
Rifamycin SV	6998-60-3	80% ACN:20% Formate (10 mM)	1	239	25	5	insoluble	697.8	1.8
Rotenone	83-79-4	90% ACN:10% Formate (0.1mM):0.1% TFA	0.5	220	13	4.1	0.17mg/L	394.4	
Shikimic Acid	138-59-0	2.5% Methanol:97.5% Water:0.1% TFA	1	213	15			174.15	
Sodium Perchlorate Monohydrate	7791-07-3	5% Methanol: 95% Water	0.5	210	10	2.2	soluble	140.45	
Sodium Valproate	1069-66-5	90% ACN:10% Water:0.1% TFA	1	260	19			166.19	
Thimerosal	54-64-8	60% Methanol:40% 10mM Ammonia Formate	1.25	220	12	-1.88	100mg/mL	404.82	
Triadimefon	43121-43-3	40% Methanol:60 % Water	0.5	225	18	2.77	7.15mg/L	293.75	
Valproic Acid	99-66-1	5% Methanol:95% Water:0.1% TFA	0.5	224	13			144.21	
Verapamil	52-53-9	55% Methanol:45% Water:0.1% TFA	1	220	15	3.79	83mg/mL	454.60	8.92

1.2 Compounds Insufficiently Soluble in the BBB Assay

We were unable to measure the transfer of four compounds due to the low solubility in the BBB assay even when dosing with 5% DMSO. These compounds are listed in **Table S2**.

Table S2: Compounds Insufficiently Soluble in the BBB Assay

Compound	CASRN
Di-(2-ethylhexyl) phthalate (DEHP)	117-81-7
Fenofibrate	49562-28-9
Ritonavir	155213-67-5
Rotenone	83-79-4

1.3 Compounds Unmeasurable in the BBB Assay

We were unable to measure the transfer of eight compounds due to kinetics that were too fast, metabolism, or degradation under the conditions of the BBB assay. These compounds are listed in **Table S3**.

Table S3: Fast and/or Metabolized Compounds

Compound	CASRN
Acrolein	1 07-02-8
Diethylacetal-Acrolein	3054-95-3
Acrylamide	79-06-1
Methoxychlor	72-43-5
Methoxyacetic Acid	625-45-6
Sodium Perchlorate Monohydrate	7791-07-3
Triadimefon	43121-43-3
Sodium Valproate	1069-66-5

2 Mathematical Derivations

2.1 Derivation of the calculation of uptake half-lives for acute exposure

Using our measured P_{app}^{AB}, we estimate the unidirectional uptake half-lives in a standard individual using HTTK-R and HTTK-R F_{ub} data. HTTK-R includes data on 11 of our compounds.

$$\frac{dC}{dt} = -F_{ub}A_{BBB}P_{app}[C] \tag{1}$$

Dimensional analysis of the above equation:

$$\frac{mol}{sec} = -(\cancel{unitless}) \cdot (\cancel{m^2}) \cdot (\frac{\cancel{cm}}{sec} \cdot \frac{\cancel{m}}{100\cancel{cm}}) \cdot (\frac{mol}{\cancel{L}} \cdot \frac{1000\cancel{L}}{\cancel{m^3}}) \tag{2a}$$

$$\frac{mol}{sec} = -10 \cdot \frac{mol}{sec} \tag{2b}$$

Where C is the mass of the compound, $[C]$ is the concentration of the compound in serum, F_{ub} is the unbound fraction of the compound in the blood and $A_{brain\ capillary}$ is the surface area of the brain capillaries, which was taken as $20m^2$ for humans, and P_{app} is our measured P_{app}^{AB}. The product $F_{ub} \cdot A_{BBB} \cdot P_{app}$ can be taken as a first-order rate constant (k) from which a half-life can be calculated using;

$$t_{1/2} = \frac{ln(2)}{k} \tag{3}$$

For Acetaminophen, our measured P_{app}^{AB} was $3.7x10^{-6}$cm/sec, F_{ub} from HTTK-R is 0.94, and A_{BBB} is taken as 20 m^2 as a typical value for an adult human. From these data the forward rate constant (Ap→ Bl) k is;

$$k = 10 \cdot 0.94 \cdot 20m^2 \cdot 3.7x10^{-6} cm/sec \quad (4a)$$
$$= 6.96x10^{-4} sec^{-1} \quad (4b)$$
$$t_{1/2} = ln(2)/6.96x10^{-4} sec^{-1} \quad (4c)$$
$$= 996 sec = 16.6 min \quad (4d)$$

2.2 Rational for not using f_{ub} in the calculation of $[C_{ss,brain}]$

In the paper, we calculate the steady-state concentration of compounds in the brain, $[C_{ss,brain}]$, based on the steady-state concentration of the compound calculated by HTTK-R in the serum, $[C_{ss,serum}]$, and the efflux ratio during continuous (chronic) exposure using the equation:

$$[C_{ss,brain}] = [C_{ss,serum}] \cdot \frac{P_{app}^{AB}}{P_{app}^{BA}} \quad (5)$$

In this calculation we do not include the fraction unbound of the compound in the serum, F_{ub}, or the brain ($F_{ub,brain}$). We did this for two reasons, first the $F_{ub,brain}$ is rarely known and it seems that the only logical assumption is that it is similar to the serum F_{ub}. With this assumption, the equation above, when including F_{ub} becomes:

$$F_{ub} \cdot [C_{ss,brain}] = F_{ub,brain} \cdot [C_{ss,serum}] \cdot \frac{P_{app}^{AB}}{P_{app}^{BA}} \quad (6)$$

where $F_{ub} = F_{ub,brain}$. Since the goal of this analysis is to compare estimates of the relative concentrations of the compound in the serum versus the brain, the inclusion of F_{ub} is unnecessary. The F_{ub} values will change the absolute concentrations but not the relative concentrations in the two compartments.

3 HTTK-R Data

Table S4 gives the Caco-2 values extracted from the HTTK-R data set for our compounds. **Table S5** contains the compound-specific data extracted from the HTTK-R data tables. Finally, the entire data set extracted from the HTTK-R package is shown in **Table S6**.

Table S4: BBB P_{app} Values and Caco-2 Values ($x10^{-6} cm/sec$) From HTTK-R Data Set

CASRN	Compound	$P_{app}AB$	+2SD	$P_{app}BA$	-2SD	Caco-2	lower 95%	upper 95%
52-53-9	Verapamil	0.57	0.02	1.0	0.14	21.1	9.36	47.5
103-90-2	Acetaminophen	3.7	0.64	2.28	0.49	23.4	10.4	52.8
19216-56-9	Prazosin	3.72	0.78	7.0	1.8	43.6	19.4	98.4
50-23-7	Hydrocortisone	4.81	2.47	1.26	0.6	16.8	7.47	37.9
298-46-4	Carbamazepine	12.15	1.33	10.87	0.41	39.8	22.2	71.6
15686-71-2	Cephalexin	12.69	3.31	25.81	2.47	0.151	0.0298	0.769
51-28-5	2,4-Dinitrophenol	13.45	0.65	11.3	1.45	60.1	33.4	108
57-41-0	Phenytoin	15.14	2.79	14.55	2.22	45.4	25.2	81.6
58-08-2	Caffeine	16.61	1.67	10.6	2.28	39.7	22.1	71.3
67-20-9	Nitrofurantoin	18.33	0.64	23.53	1.28	2.28	1.27	4.09
414864-00-9	Belinostat	18.85	25.95	0.07	0.05			
150-76-5	Methoxyphenol	54.78	11.09	37.61	10.22	51.7	28.7	92.9

Table S5: Estimated Half Lives and $C_{ss,brain}$

CASRN	Compound[a]	P_{app}^{AB} $(x10^{-6} cm/sec)$	Human F_{ub}[b]	Acute Uptake k (min^{-1})[e]	Acute $t_{1/2}$ (min)	$[C_{ss,serum}]^b$ (μM)	Efflux Ratio[b]	$[C_{ss,brain}]$ (μM)[f]
89-25-8	Edaravone	0.41	0.89	0.0044	158.30	0.456	0.610	0.747
52-53-9	Verapamil	0.57	0.1	0.0007	1013.4	0.221	1.754	0.126
103-90-2	Acetaminophen	3.7	0.9398	0.0417	16.611	1.695	0.616	2.751
19216-56-9	Prazosin	3.72	0.06	0.0027	258.79	2.530	1.882	1.345
50-23-7	Hydrocortisone	4.81	0.2	0.0115	60.044	0.327	0.262	1.248
84-66-2	Diethyl Phthalate	7.71	0.19	0.0176	39.431	0.095	0.935	0.101
298-46-4	Carbamazepine	12.15	0.5141	0.0750	9.2474	0.945	0.895	1.056
5786-21-0	Clozapine	13.37	0.05	0.0080	86.406	1.411	0.894	1.579
51-28-5	2,4-Dinitrophenol	13.45	0.02723	0.0044	157.72	76.050	0.840	90.520
54910-89-3	Fluoxetine	14.4	0.13	0.0225	30.856	1.353	1.399	0.967
57-41-0	Phenytoin	15.14	0.1624	0.0295	23.493	4.707	0.961	4.898
58-08-2	Caffeine	16.61	1.0	0.1993	3.4776	1.334	0.638	2.090
67-20-9	Nitrofurantoin	18.33	0.415	0.0913	7.5934	3.938	1.284	3.068
414864-00-9	Belinostat	18.85	0.06	0.00023	51.1	6.043	0.004	1627.3
80-05-7	Bisphenol A	22.89	0.0385	0.0106	65.545	0.943	1.244	0.758
10265-92-6	Methanidophos	56.07	0.396	0.2664	2.6015	.	0.326	.
99-66-1	Valproic Acid	>500[d]	0.214	1.2840	0.5398	7.256	0.235	30.903
43121-43-3	Triadimefon	>500[d]	0.1142	0.6852	1.0116	0.366	0.299	1.224

[a] Compounds are sorted by ascending P_{app}^{AB}.
[b] From HTTK-R.
[c] $P_{app}^{BA}/P_{app}^{AB}$.
[d] Assumed to be 500 for subsequent calculations.
[e] Column was calculated using Equation 3 in the main paper.
[f] Column was calculated using Equation 4 in the main paper.

Table S6: HTTK-R Data Table[a]

CASRN	Compound	Human.Funbound .plasma	Caco-2 avg[b]	Caco-2 lower 95%[b]	Caco-2 upper 95%[b]	DTXSID[c]	logP	Molecular Weight	pKa_Accept	pKa_Donor	Human .Clint.AVG[d]	Human.Funbound .plasmaAVG	Human.Rblood 2plasma	HTTK Css,serum (μM)[e]
89-25-8	Edaravone					9021130	1.783	174.2		14.6	2	0.89		0.4555
52-53-9	Verapamil	0.1	21.1	9.36	47.5	9041152	3.791	454.6	9.68		12.7	0.1	0.77	0.2209
103-90-2	Acetaminophen	0.940	23.4	10.4	52.8	2020006	0.462	151.2		9.46	0.360	0.940	1	1.695
19216-56-9	Prazosin	0.06	43.6	19.4	98.4	4049082	2.363	383.4	7.24,0.94		3.349	0.06	0.7	2.53
50-23-7	Hydrocortisone	0.2	16.8	7.47	37.9	7020714	1.611	362.5			8.567	0.2		0.3268
84-66-2	Diethyl Phthalate	0.19				7021780	2.444	222.2			42.5	0.19		0.0948
298-46-4	Carbamazepine	0.514	39.8	22.2	71.6	4022731	2.449	236.3			2.375	0.514	1.06	0.9446
5786-21-0	Clozapine	0.05				5022855	3.23	326.8	7.35,3.92		8.567	0.05	0.87	1.411
51-28-5	2,4-Dinitrophenol		60.1	33.4	108	0020523	1.672	184.1		4.35	0	0.0272		76.05
54910-89-3	Fluoxetine	0.13				7023067	4.051	309.3	9.8		1	0.13	0.83	1.353
57-41-0	Phenytoin	0.162	45.4	25.2	81.6	8020541	2.364	252.3		8.49	0.818	0.162	0.887	4.707
58-08-2	Caffeine	1	39.7	22.1	71.3	0020232	-0.071	194.2			0.286	1	1.08	1.334
67-20-9	Nitrofurantoin	0.415	2.28	1.27	4.09	7020972	-0.469	238.2		8.23,13.7	0	0.415	0.76	3.938
80-05-7	Bisphenol A	0.038				7020182	3.32	228.3		9.78,10.4	19.9	0.038	0.770	0.9432
10265-92-6	Methanidophos					6024177	-0.799	141.1			0	0.396		
99-66-1	Valproic Acid		29.2	12.9	65.7	6023733	2.748	144.2		5.14	0.437	0.214	0.74	7.256
43121-43-3	Triadimefon	0.114				3023897	2.771	293.8	1.93		0	0.114		0.3656
117-81-7	Bis(2-ethylhexyl) phthalate					5020607	7.525	390.6			0	0.046		4700
49562-28-9	Fenofibrate	0.00815				2029874	4.943	360.8			563	0.008		0.0880
414864-00-9	Belinostat	0.06	.	.	.	60194378	2.001	318.4		.	0.782	0.06	.	6.043

Table S6 Notes

[a] For the column definitions, see the HTTK-R documentation.

[b] $x10^{-6} cm/sec$.

[c] Prepend "DTXSID" to the number given.

[d] $uL/min/(10^6$ hepatocytes).

[e] HTTK $[C_{ss,serum}]$, for a dose of 1mg/kg/day via continuous infusion, in uM, calculated for venous plasma.
